# Roles and therapeutic implications of m6A modification in cancer immunotherapy

**DOI:** 10.3389/fimmu.2023.1132601

**Published:** 2023-03-07

**Authors:** Juan Pan, Tuxiong Huang, Zhenjun Deng, Chang Zou

**Affiliations:** ^1^ National Center for International Research of Bio-targeting Theranostics, Guangxi Key Laboratory of Bio-targeting Theranostics, Collaborative Innovation Center for Targeting Tumor Diagnosis and Therapy, Guangxi Talent Highland of Bio-targeting Theranostics, Guangxi Medical University, Nanning, Guangxi, China; ^2^ Department of Clinical Medical Research Center, The 2nd Clinical Medical College (Shenzhen People’s Hospital) of Jinan University, The First Affiliated Hospital of Southern University of Science and Technology, Shenzhen, China; ^3^ Guangdong Provincial Key Laboratory of Regional Immunity and Diseases, Department of Pharmacology and International Cancer Center, Shenzhen University Health Science Center, Shenzhen, China; ^4^ Department of Dermatology, The Second Clinical Medical College, Jinan University (Shenzhen People’s Hospital), Shenzhen, China; ^5^ The First Affiliated Hospital, Jinan University, Guangzhou, China; ^6^ Shenzhen Public Service Platform On Tumor Precision Medicine and Molecular Diagnosis, The Second Clinical Medical College of Jinan University, Shenzhen People’s Hospital, Shenzhen, China

**Keywords:** m6A modification, cancer, immune checkpoints, m6A-regulator inhibitors, immunotherapy

## Abstract

Recent studies have demonstrated that N6-methyladenosine (m6A), the most abundant, dynamic, and reversible epigenetic RNA modification in eukaryotes, is regulated by a series of enzymes, including methyltransferases (writers), demethylases (erasers), and m6A recognition proteins (readers). Aberrant regulation of m6A modification is pivotal for tumorigenesis, progression, invasion, metastasis, and apoptosis of malignant tumors. Immune checkpoint inhibitors (ICIs) has revolutionized cancer treatment, as recognized by the 2018 Nobel Prize in Medicine and Physiology. However, not all cancer patients response to ICI therapy, which is thought to be the result of intricate immune escape mechanisms. Recently, numerous studies have suggested a novel role for m6A epigenetic modification in the regulation of tumor immune evasion. Herein, we review the relevant mechanisms of m6A regulators in regulating various key signaling pathways in cancer biology and how m6A epigenetic modifications regulate the expression of immune checkpoints, opening a new window to understand the roles and mechanisms of m6A epigenetic modifications in regulating tumor immune evasion. In addition, we highlight the prospects and development directions of future combined immunotherapy strategies based on m6A modification targeting, providing directions for promoting the treatment outcomes of immune checkpoint inhibitors.

## Introduction

Post-translational modifications participate in many biological processes including disease progression, and are emerging as important regulators in tumors. RNA methylation modifications, the dominant RNA modification forms, including N^6^-methyladenosine (m6A), N1-methyladenosine (m1A), N7-methylguanidine (m7G), 5-methylcytidine (m5C), 2′-O-methylation (Nm), and 5-hydroxymethylcytidine (1) have been reported to influence diverse biological processes. Normal body operations can be ensured when these modifications are performed correctly (2). Among various types of epigenetic modifications, N6-methyladenosine (m6A) is the most prevalent internal modification in eukaryotic cells (3). N6-methyladenosine (m6A) involves methylation of the sixth nitrogenous base of adenylated RNA/DNA by the addition of a methyl group (4), which is extensively present in mRNA (5, 6), long noncoding RNAs (lncRNAs) (7), microRNAs (miRNAs) (8), small nuclear RNAs (snRNAs) (9), small nucleolar RNAs (snoRNAs) (10), and ribosomal RNAs (rRNAs) (11) (12). Modern molecular techniques allow the study of m6A modifications in the transcriptome with high efficiency. Recently, continuous progress in next-generation sequencing (NGS) technology has enabled the mapping of the landscape of m6A in the transcriptome (termed “epitranscriptome”) in 2012 (3). This has also revealed that m6A modification widely occurs in a highly conserved consensus sequence known as RRACH (where R=A or G, H=A, C or U) (13, 14) and deposits not only around the stop codon but also in the coding sequence (long internal exon) and 3′ untranslated region (UTR) (15, 16). Similar to DNA and protein methylation, RNA can be methylated and demethylated with the help of methyltransferases and demethylases, which regulate the mRNA life cycle dynamically and reversibly (17).

**Figure 1:**
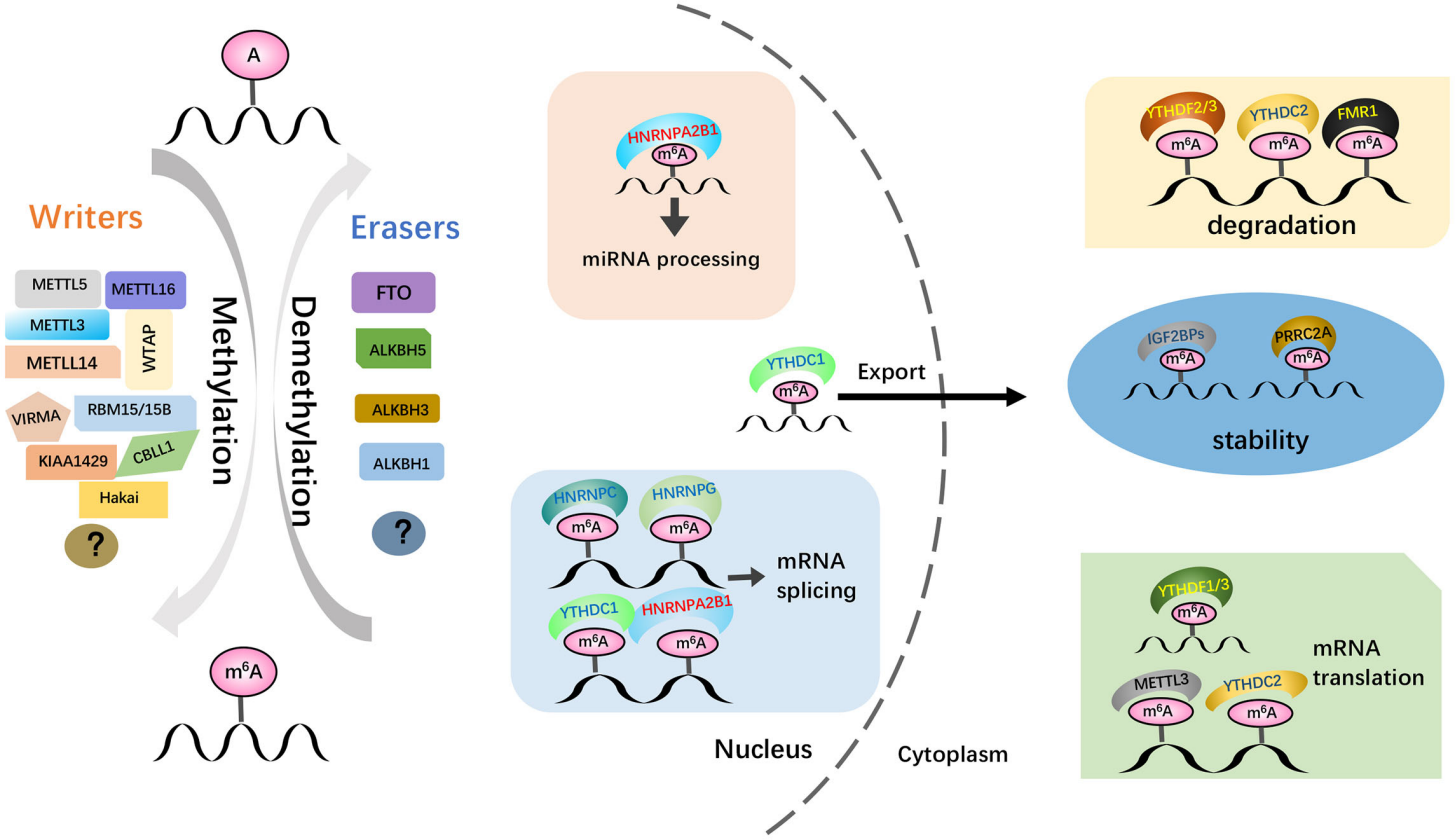
The dynamic and reversible m6A RNA modification is regulated by “writers”, “erasers.”, and “readers”. M6 A methylation is installed by the methyltransferase complex comprising the core catalytic subunit METTL3/METTL14/WTAP and other regulatory proteins. The erasers are composed of FTO, ALKBH5, and ALKBH3, removing the methyl group by demethylation. The function of m6A is mediated by m6A ‘reader’, recognizing m6A marks to activate downstream regulatory pathways, which mediate various functions of RNA, such as RNA splicing, export, decay, stabilization and translation. And the reader proteins mainly include YTHDF1/2/3, YTHDC1/2, the IGF2BP family, HNRNPs (HNRNPA2B1, HNRNPC and HNRNPG), eIF3, PRRC2A, and FMR1.

m6A modification is orchestrated by coordinated behavior of three homologous factors, namely, “writers,” (methyltransferase), “erasers” (demethylase), and “readers” (recognition), which can install, remove, or recognize m6A, respectively, affecting multiple metabolisms of m6A-containing RNA, including mRNAs and noncoding RNAs (lncRNA, miRNAs, and circRNA) (18) (**Figure 1**). Writers of m6A methylation consist of the core catalytic subunits METTL3/METTL14, WTAP, the major components of the methyltransferase complex (MTC) in the nucleus, and their cofactors such as HAKAI, VIRMA (KIAA1429), ZC3H13, and RBM15/15B, catalyzing the m6A modification of distinct RNAs (19). The main demethylases (“erasers”) include FTO and ALKBH5, which remove m6A modification from RNA, thus ensuring that m6A modification is an invertible and dynamic process (20). In addition, m6A-modified RNAs are required for “readers,” which recognize and bind to certain motifs mastering the modified RNA fate, and this mainly includes the YTH domain family of proteins (21) and IGF2BP proteins (22). Thus, m6A modification plays an important role in mediating fundamental pathological and physiological metabolic processes, processing (8), translation (23), and stability of RNA (24). Several studies have shown that this modification has a significant effect on cellular biological functions (bioprocesses), including stem cell self-renewal and differentiation, heat shock or DNA damage response, tissue development, and maternal-homeostasis transformation (6). For instance, FTO has been reported to promote HCC stemness (25). m6A modification participates in the pathogenesis and progression of various cancers, such as lung adenocarcinoma, acute myeloid leukemia (AML), ovarian cancer, hepatocellular carcinoma (HCC), gastric cancer, colorectal cancer (CRC), and melanoma (26). Furthermore, m6A modification was not only found in tumors but also in ischemic diseases (27). Meanwhile, aberrant m6A modification plays vital roles in diverse tumor immunity processes, including the infiltration, activation, and effector functions of infiltrated immune cells in the tumor microenvironment (TME) (28), suggesting its crucial roles in cancer immunotherapy.

**Figure 2:**
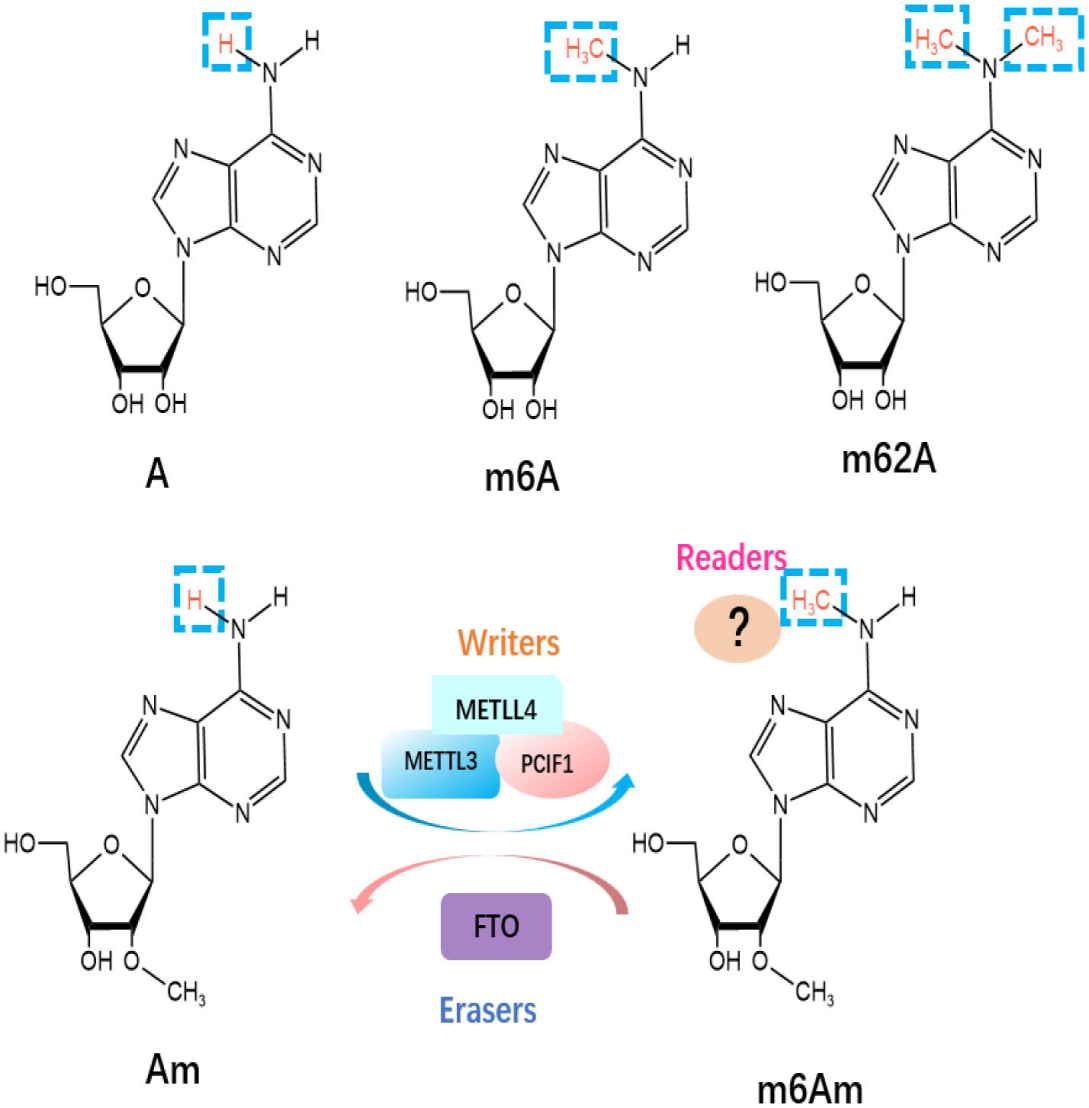
Molecular structures of RNA methylations. The m6Am modification is methylated by “writers”, including METTL3, METTL4 and PCIF1, and eliminated by “eraser” FTO.

Besides m6A, m6Am and m62A are two other common epigenetic modifications identified as members of the m6A “family” in mRNA (**Figure 2**). N6, N6-Dimethyladenosine (m62A), a conserved modification discovered in 18S rRNA and also found in tRNA from mycobacterium bovis Bacille Calmette-Guérin (29), was shown to play a pivotal role in ribosome biogenesis (30). m6Am is located at the first transcribed nucleotide, near the cap 7-methyl Guanosine mRNA(m7G) cap structure, which is installed by the methyltransferases [i.e., PCIF1 (31), METTL3, and METTL4 (32)] (33), as there may be a link to an immunogenic role (34). Wang’s group has recently reported the vital role of PCIF1 in regulating the sensitivity to anti-PD-1 therapy in CRC. To be more specific, depletion of Pcif1 in CRC cells enhanced the effects of anti‐PD‐1 treatment in immunocompetent mice by increasing IFN-γ, TNF-α, and decreasing TGF-β levels, which recruited tumor‐infiltrating natural killer cells in TME (35). While there is no data concerning m62A in the literature concerning ICI modulation, AlkBH5 is known to demethylate m62A (36) and has been linked to modulating anti-PD1 therapy response (37). As a reversible RNA methylation, m6Am is removed by the demethylase FTO (38, 39). FTO is also an m6A eraser, but it appears the physiologically relevant substrate may be m6Am, not m6A (39). Liu et al. has compared the distribution of m6A versus m6Am across human and mouse tissues, revealing that the m6A and m6Am methylomes of brain tissues are highly specific and the major determinant of methylome is species type instead of tissue type (40). Some older methods confuse the two marks, and so new methods have been developed to distinguish between m6A and m6Am (41). This means that due to the recent realization and distinction between m6A and m6Am, some current and previous experimental work directed at m6A may actually be directed at m6Am residues. m6A enzymes are associated with RNA metabolic processes, including RNA splicing, export, translation, degradation. However, m6Am has been reported to perform different biological functions, including RNA splicing (32), snRNA biogenesis (42), mRNA stability (39, 43), and cap-dependent translation of their downstream targets (44). Furthermore, Liu et al. has revealed that m6Am was negatively correlated with protein expression level (40).

**Figure 3:**
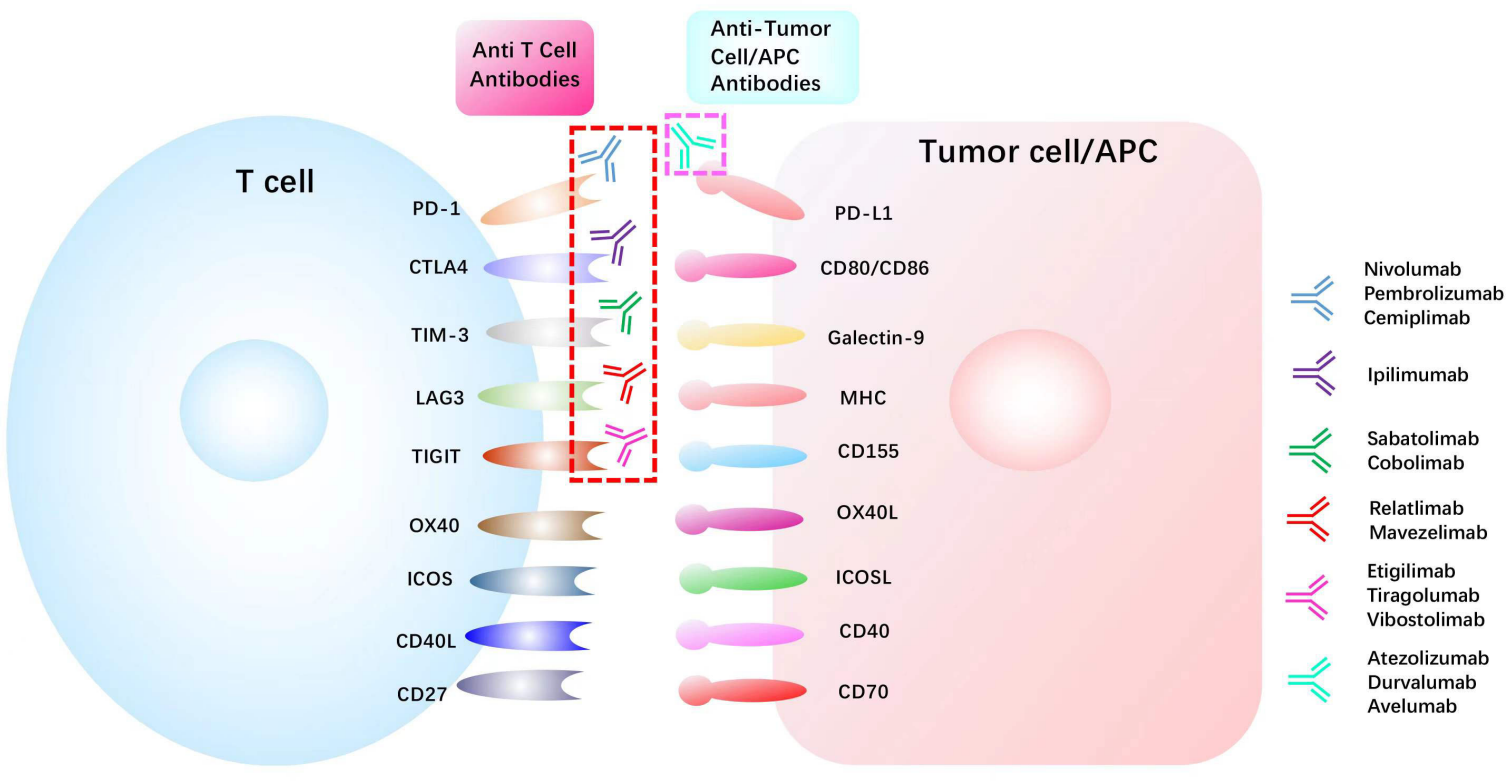
List of common immune checkpoints and its related inhibitors in clinical trials PD1, programmed cell death 1; PDL1, programmed cell death 1 ligand 1; CTLA4, cytotoxic T lymphocyte antigen 4; TIM-3, T cell immunoglobulin mucin receptor 3;CD40L, CD40 ligand; ICOS, inducible T cell co-stimulator; ICOSL, inducible T cell co-stimulator ligand; LAG3, lymphocyte activation gene 3 protein; MHC, major histocompatibility complex; OX40L, OX40 ligand; TIGIT, T cell immunoreceptor with Ig and ITIM domains.

Current cancer treatments mainly consist of surgery, chemoradiotherapy, targeted therapy, and immunotherapy. Among these therapies, immunotherapy has become the mainstream of modern cancer treatment (45) by manipulating the self-immune system to recognize and attack cancer cells and has achieved unprecedented success in clinical trials. Especially for lung cancer, as the most common cancer worldwide, the prognosis of advanced NSCLC (46) and extensive‐stage small‐cell lung cancer (SCLC) (47) was significantly improved with the administration of immunotherapy. Up to now, two PD1 inhibitors Nivolumab and Pembrolizumab and one PD-L1 inhibitor Atezolizumab have been approved as standard treatment options for NSCLC patients (48). Immune checkpoint inhibitors, such as atezolizumab, avelumab, and durvalumab, have been used to restore the anti-tumor immunity (**Figure 3**) and have shown to be effective in a wide range of tumors. Nivolumab plus ipilimumab has demonstrated durable overall survival benefit in melanoma (49), renal cell carcinoma (50), and NSCLC (51). However, in most cases, only a small number of patients can benefit, and the response rate and efficacy of current immunotherapies remain unsatisfactory (**Table 1**). The complex immune escape mechanisms in tumors is one of the most critical reasons. Therefore, further exploration of the mechanisms regulating changes in immune checkpoints and escape mechanisms is justified to improve the effects of current immunotherapy. In recent years, compelling evidence has indicated that m6A methylation can regulate tumor immunity (63).

**Table 1:** Efficacy of different immune checkpoint inhibitors in various cancer trials.

Cancer type	ICIs used in trial	Trial population	ORR
**CRC**	Pembrolizumab, Nivolumab, Atezolizumab, Ipilimumab	MSI-unselected	8~40% (52)
		MSI‐H/dMMR	28~55% (52)
		MSS/pMMR	3~13% (52)
**NSCLC**	Pembrolizumab, Nivolumab, Atezolizumab, Avelumab	unselected	16.7~22.4% (53)
		PD-L1^high^	24%~45.2% (54)
**SCLC**	Pembrolizumab Atezolizumab	unselected	~19% (53)
**Melanoma**	Pembrolizumab, Nivolumab, Ipilimumab	unselected	24~60% (53, 55)
**cSCC**	Cemiplimab	unselected	~50% (56)
**mRCC**	Nivolumab, Ipilimumab	unselected	~40% (57)
**Urothelial** **cancer**	Atezolizumab, Durvalumab, Avelumab, Nivolumab, Pembrolizumab	unselected	14.5~24% (58)
**Gastroesophageal cancer**	Nivolumab, Pembrolizumab, Durvalumab	unselected,	10~19% (59)
		PD-L1^+^	22-30% (60)
**HCC**	Nivolumab, Pembrolizumab	unselected	15-20% (61)
**TNBC**	Atezolizumab	unselected	53% (62)

ICIs, Immune Checkpoint Inhibitors; ORR, overall or objective response rate; MSI, microsatellite instability; MSI-H, MSI-high; dMMR, mismatch repair deficient; MSS, microsatellite stable; pMMR, mismatch repair proficient; NSCLC, Non-small cell lung cancer; SCLC, Small cell lung cancer; cSCC, Cutaneous squamous cell carcinoma; mRCC, metastatic renal cell carcinoma; HCC, Hepatocellular carcinoma; TNBC, triple-negative breast cancer.

In this review, we elaborated on the effects of m6A epitranscriptome modification on tumor immunotherapy for different types of cancer and focused on the recent progress on the impact of m6A regulators in canonical signaling pathways of various cancers and the mechanisms of m6A epigenetic modifications regulating cancer immune checkpoint inhibitors (ICIs) response. We also highlight the potential clinical applications and future directions of m6A modification in cancer immunotherapy among patients.

### m6A-dependent functions of m6a regulators in canonical pathways for cancer regulation

**Figure 4:**
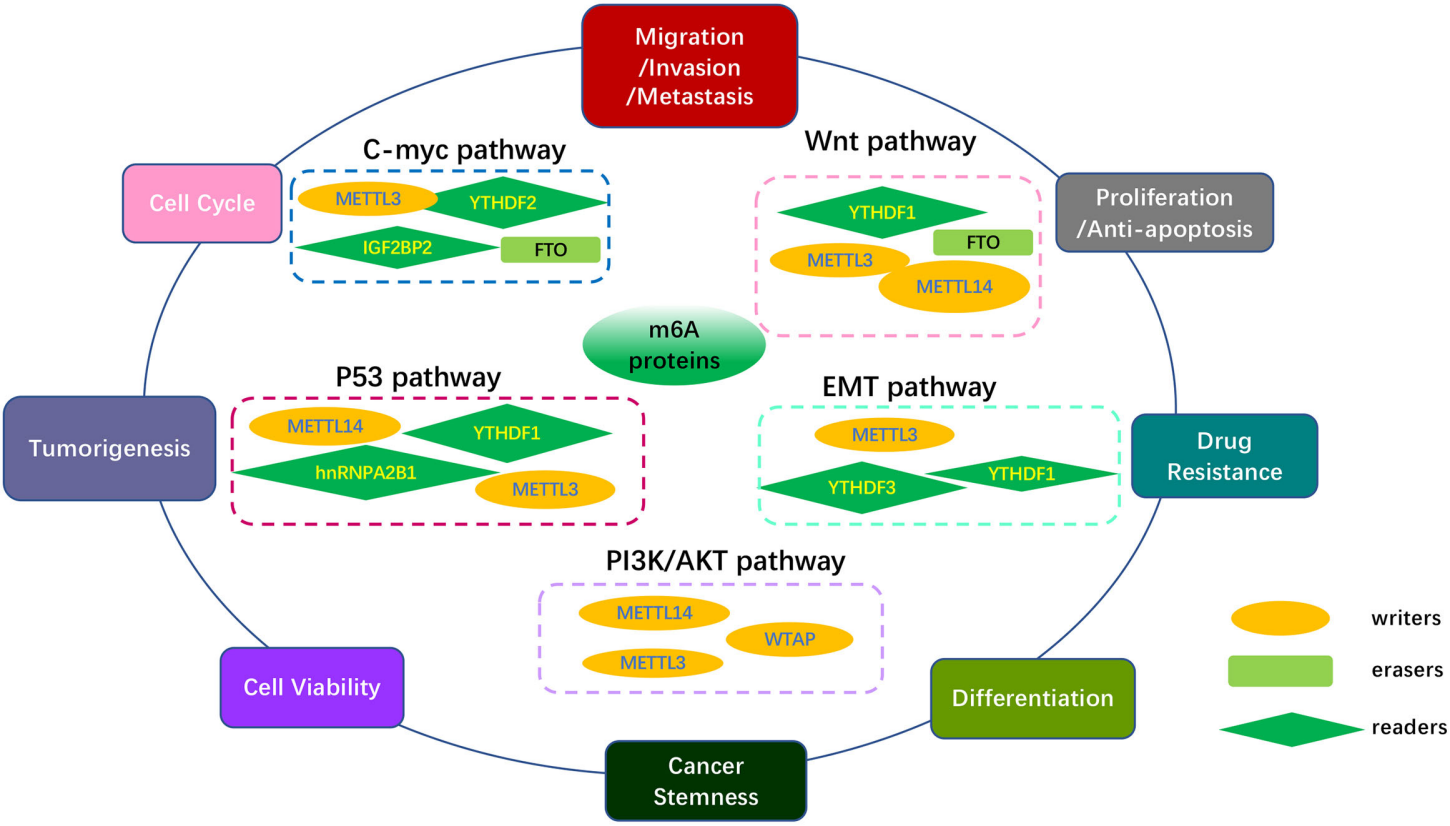
Overview of multiple functions of m6A regulators in various cancers mediated by some signaling pathways. The core pathways regulate many biological functions of tumors, including tumor occurrence, migration, proliferation, apoptosis, differentiation metastasis, multidrug resistance, treatment response and cancer development. The MYC pathway, Wnt/β-Catenin pathway, PI3K/AKT/mTOR pathway, p53 pathway, EMT pathway, and other pathways are included in the key cancer pathways.

Over the past few years, extensive studies have suggested that m6A epigenetic modification contributed to cancer progression (64). Significant progress has been made in understanding the critical role of m6A regulatory proteins involved in many canonical pathways, such as C-MYC, Wnt/β-catenin, PI3K/AKT/mTOR, p53, and epithelial-mesenchymal transition (EMT), which may be potential therapeutic signaling pathways due to their key role in tumor occurrence, migration, proliferation, apoptosis, metastasis, drug resistance, and treatment response. The roles of m6A regulators in modulating core genes and key pathways in various cancers are summarized in **Table 2** and **Figure 4**.

**Table 2:** The role of m6A regulators in various cancers *via* the key pathways.

Pathway	m6A regulator	Cancer	Function
C-myc pathway	METTL3	Lung cancer	promote growth and migration (65)
	METTL3	Bladder cancer	promote cell proliferation, invasion and survival (66)
	METTL3	Oral squamous cell carcinoma, Colorectal cancer, Prostate carcinoma	promote growth, invasion, migration and progression (67) (68) (69),,
	METTL3	Gastric cancer	promote proliferation and metastasis (70)
	METTL3	Acute myeloid leukemia	inhibit diferentiation and increase proliferation (71)
	FTO	Colorectal cancer	inhibit apoptosis and improve cell proliferation, migration, and invasion (72)
	IGF2BP2	Thyroid cancer	promote proliferation, invasion, migration and anti-apoptosis (73)
	YTHDF2	Glioblastoma	support glioblastoma stem cells viability (74)
Wnt signaling pathway	YTHDF1	Gastric cancer	promote gastric carcinogenesis (75)
	FTO	Endometrial cancer	promote invasion and metastasis (76)
	YTHDF1	Colorectal carcinoma	promote tumorigenicity and cell cycle (77)
	METTL3	Hepatocellular carcinoma	accelerate development (78)
		Hepatocellular carcinoma	induce sorafenib resistance (79)
	METTL3	Colorectal carcinoma	promote the stemness and chemoresistance (80)
	METTL3	Nasopharyngeal carcinoma (NPC)	promote cisplatin resistance (81)
	METTL14	Breast cancer	promote stemness and progression (82)
PI3K/AKT/mTOR pathway	WTAP	Osteosarcoma	promote proliferation and metastasis (83)
	METTL14	Colorectal carcinoma	inhibit Colorectal carcinoma malignant process (84)
	METTL3	Ovarian cancer	promote ovarian Cancer development (85)
	METTL3	Retinoblastoma	promote Retinoblastoma progression (86)
p53 pathway	METTL3	Colorectal carcinoma	promote multidrug resistance (87)
	YTHDF1 and HNRNPA2B1	Melanoma	promote the development (88)
	METTL14	Pancreatic cancer	promote the growth and metastasis (89)
	METTL3	Breast cancer	promote the proliferation (90)
EMT signaling pathway	METTL14	Colorectal carcinoma	mediate EMT process (84)
	METTL3	Gastric cancer	accelerate the EMT (91)
	METTL3	Hepatocellular carcinoma	promote the EMT (92)
	YTHDF3	Hepatocellular carcinoma	facilitate migration, invasion, and EMT (93)
	METTL3	Lung cancer, Ovarian cancer, Colorectal carcinoma	accelerate the EMT and promote the development (94) (95) (96),,
Other signaling pathways			
MAPK signaling pathway.	METTL3	Colorectal carcinoma	promote metastasis (97)
P38/ERK	METTL3	Colorectal carcinoma	suppress proliferation, migration and invasion (98)
ERK1/2 and STAT3 pathways	HNRNPA2B1	Breast cancer	promote the tumorigenicity, and decrease apoptosis (99)
BCL-2	METTL3	Breast cancer	accelerate proliferation, decrease the apoptosis (100)

MYC is one of the most commonly activated oncogenes that mediate cancer initiation and progression. It has been reported to be involved in almost every aspect of the oncogenic process, including differentiation, proliferation, apoptosis, and metabolism, as a molecular hallmark of cancer (101). In addition, it regulates the expression of two immune checkpoints, CD47 and PD-L1, by directly binding to their promoters (102), allowing MYC to be confirmed as a target for immunotherapy resistance. Many studies have revealed that the effect of m6A on various cancers relies on changes in the C-myc pathway or its related genes. As shown in **Table 2**, m6A regulators, including METTL3, FTO, and IGF2BP2, function as tumor promoters through the C-myc pathway in an m6A dependent manner, promoting growth, invasion, migration, and progression in various tumors such as lung cancer (65), bladder cancer (66), oral squamous cell carcinoma (OSCC) (67), CRC (68), prostate carcinoma (PCa) (69), and Gastric Cancer (70).

Many studies have shown that Wnt/β-catenin signaling contributes to the primary resistance to immunotherapy by influencing tumor-cell functions and immune surveillance (103). Fu et al. revealed that β-catenin in dendritic cells (DCs) plays a positive role in maintaining CD8+ T cells through the regulation of interleukin-10 (104), which is considered to be relevant to immune evasion. In addition, many studies have shown that β-catenin activation is associated with Treg infiltration (105), T-cell rejection, and resistance to anti-PD-L1/anti-CTLA-4 immunotherapy (106). It has also been reported that the crosslink between cancer cells and tumor-associated macrophages (TAMs) is realized through Wnt/β-catenin signaling (107). Thus, we conclude that inhibiting the Wnt/β-catenin pathway may hold immense potential as a possible adjuvant for immunotherapy. The Wnt/β-catenin signaling pathway can be regulated by m6A modifications, and then facilitate chemoresistance in various cancers. For example, in nasopharyngeal carcinoma (NPC), TRIM11 is upregulated by METTL3-mediated m6A modification, which modulates β-catenin signaling, thus promoting cisplatin resistance (81).

In addition to the above-mentioned two pathways, m6A affects cancer pathogenesis and progression *via* other classical pathways, including the phosphatidylinositol-3-kinase (PI3K)/AKT/mammalian target of rapamycin (mTOR), p53, EMT, mitogen-activated protein kinase (MAPK), and p38/extracellular signal-regulated kinase (ERK). It has been reported that activation of the PI3K/AKT/mTOR pathway is relevant to PD-L1 expression and tumor immune microenvironment (108), implying a novel indication for cancer immunotherapy. A study reported that AKT inhibitors could preferentially suppress Tregs and enhance the number of CD8+ T cells, leading to the inhibition of tumor progression. Moreover, inhibiting PI3K/AKT/mTOR can promote the secretion of immunosuppressive cytokines (109) and infiltration of myeloid-derived suppressor cells (MDSCs) (110), which would enhance the anti-tumor immune response. The tumor suppressor p53 acts as a transcription factor, with mutations present in approximately 50% of all invasive tumors (111). Mutant p53 strengthened neoantigenesis, and patients with greater mutational burden showed a better response to anti-PD-1 therapy in many clinical studies, appearing to serve as a predictor of ICI treatment efficacy (112). EMT is characterized by losing their epithelial cell identity marker E-cadherin and acquiring features of mesenchymal marker vimentin related to salient malignant properties of the tumor, including primary tumor formation, tumor stemness, malignant progression, tumor cell migration, intravasation to the blood, and metastasis (113, 114). EMT has also been reported to induce local immunosuppression in the TME, thereby destroying immunosurveillance and inducing resistance to immunotherapy (115). As shown in **Table 2**, m6A regulators METTL3, METTL14, WTAP, YTHDF1, and HNRNPA2B1 have been involved in many aspects of various tumors as promoters through the PI3K/AKT/mTOR, p53, and EMT signaling pathways in an m6A dependent manner. Thus, the development of inhibitors/agonist-targeted m6A regulators might improve the efficacy of immunotherapy. Additionally, recent research has shown that the mTOR, Raf/mitogen-activated protein kinase (MEK)/ERK, AMP-activated protein kinase (AMPK), nuclear factor kappa B (NF-κB), and Hedgehog signaling pathways are closely related to m6A modification, which mediates the regulation of tumor phenotypes (116). Moreover, these signaling pathways have also been reported to be involved in improving T cell activation (117), regulating metabolism in T cells (118), T cell-dependent immune responses (119), and the development of immune cell homeostasis and immune response (120). Therefore, m6A modification uncovered a novel mechanism regulating cancer progression, and identification of the strong connection between m6A regulators and the above pathways might set the ground for future research and promising ICI therapy.

### m6A modification and regulation of anti-tumor immunity

Tumor immunotherapy, one of the most effective treatments for cancers, has become the mainstream modern cancer treatment (45) and has drawn extensive attention in recent years. ICIs, mainly targeting CTLA4, PD-1, and PD-L1, has achieved remarkable success in the past decades, which has improved the outcomes of patients with advanced-stage cancer. Despite the therapeutic success achieved in the combination of ICIs with other therapies, many patients might not respond or have a low response to current treatment, thus, drug resistance and relapses are common treatment difficulties found nowadays (46, 121). The lack of responsiveness demonstrates a clear need to outline the underlying mechanisms of tumor ICIs for the treatment of tumors. Complex immune escape pathways are important. Recently, many studies have suggested an important role of m6A modification in regulating tumor immune evasion. They also demonstrated that m6A epigenetic modification is highly correlated with the efficacy of ICIs by directly or indirectly affecting the expression levels of ICIs targets, including PD-1, PD-L1, CTLA4 and other checkpoints (**Table 3**), or some key signaling pathways.

**Table 3:** The functions and mechanisms of m6A modification on immune checkpoints.

Regulator	Immune checkpoint	Cancer	Function/Mechanism
METTL3	PD-L1	Non-small cell lung cancer	modify circIGF2BP3 gene and elevate its expression in an YTHDC1-m6A-dependent manner (122)
		Breast cancer	facilitate PD-L1 stability in an m6 A-IGF2BP3-dependent manner1 (123)
		Oral squamous cell carcinoma	(124)
	CD80		promote dendritic cell (DC) activation and maturation (125)
	ICOS		promote T follicular helper (TFH) cell differentiation (126)
METTL14	PD-L1	Cholangiocarcinoma	bind Siah2 mRNA in an YTHDF2-dependent pathway99
		Hepatocellular carcinoma	regulate MIR155HG relying on HuR-dependent pathway (127)
METTL3/14	PD-L1	Colorectal carcinoma Melanoma	regulate response to anti-PD-1 therapy (128)
	ICOS		promote T follicular helper (TFH) cell differentiation (129)
FTO	PD-1	Melanoma	promote growth/proliferation (130)
	PD-L1	Colon cancer	(131)
ALKBH5	PD-L1	Intrahepatic cholangiocarcinoma	the loss of ALKBH5 promote the degradation of PD-L1 in an YTHDF2-dependent manner (132)
IGF2BPs	PD-1	Lung adenocarcinoma	positively correlated with PD-1 expression (133)
IGF2BP1	PD-L1	Hepatocellular carcinoma	knockdown of IGF2BP1 downregulate the expression of PD-L1 (134)
YTHDF1	PD-L1 VISTA	Colorectal cancer	promotes the protein synthesis of PD-L1 and VISTA (135)
YTHDF1 YTHDF2	PD-L1	Non-small cell lung cancer	(136)
PCIF1		Colorectal cancer	Pcif1 knockout enhances the effects of anti-PD-1 treatment by decreasing TGF-β levels and increasing IFN-γ, TNF-α levels, and tumor-infiltrating natural killer cells *via* m6Am modifications (35)

#### m6A modification and PD-1

Mounting evidence has shown that m6A modification is always misregulated in various types of cancers and that m6A regulatory factor expression is significantly related to the expression of PD-1. Yang et al. revealed that highly expressed fat mass and obesity-associated (FTO) gene plays a carcinogenic role in melanoma growth *via* m6A RNA modification, and FTO knockdown increased m6A methylation of PD-1 through the m6A reader YTHDF2, leading to the promotion of cell growth and proliferation. Simultaneously, FTO inhibition enhances the response to anti-PD-1 therapy in mice (130). The IGF2BP family, a regulatory subunit of m6A readers, is positively correlated with PD-1 expression, demonstrating that m6A modification regulated by the IGF2BP family may have potential benefits for patients with lung adenocarcinoma treated with ICIs (133). In recent years, integrative and comprehensive genomic and molecular analyses have been established to explore potential prognostic targets predicting the response of patients with pancreatic cancer to immunotherapy. A low m6A score, based on the expression of some m6A regulators, revealed a low abundance of PD-1 and CTLA-4, suggesting the key role of m6A in clinical application in predicting the response to ICIs in patients with pancreatic cancer (137).

#### m6A modification and PD-L1

PD-L1, as a PD-1 ligand and another critical immune checkpoint protein, binds to PD-1, contributing to the immune escape of cancer cells (138). Emerging studies have shown that dysregulation of m6A-related regulators, such as methylases, demethylases, and m6A binding proteins, is pivotal in affecting PD-L1 expression. Depletion of Mettl3 and Mettl14 significantly upregulated PD-L1, and Mettl3 or Mettl14 deficient enhanced the response to anti-PD-1 treatment in pMMR-MSI-L CRC and melanoma. Notably, the growth of tumors, loss of Mettl3 or Mettl14, was inhibited analogous to multiple combinatorial immunotherapy regimens (anti-PD-1+anti-CTLA-4) (128). Except for the direct action of m6A regulators on PD-L1, the upstream genes of PD-L1 were also extensively referred to as the modification of m6A RNA methylation and then increasing or reducing PD-L1 expression. In non-small cell lung cancer (NSCLC), METTL3 mediates m6A modification of the circIGF2BP3 (hsa_circ_0079587) gene in a YTHDC1-m6A-dependent manner, which competitively upregulates PKP3 expression. PKP3 stabilizes PD-L1 protein by facilitating its deubiquitination, eventually contributing to the immune escape of NSCLC cells (122). Similar to NSCLC, in cholangiocarcinoma (CCA), METTL14 binds Siah2 mRNA in the 3′-UTR region and triggers m6A modification, promoting its degradation in a YTHDF2-dependent manner. Upon the depletion of Siah2, it increased the protein stability of PD-L1 and then inhibited T cells expansion and T-cell–mediated anti-tumor activity in CCA cells, indicating the clinical potential of the METTL14-Siah2-PD-L1–regulating axis for CCA immunotherapy (139). Furthermore, lncRNA is also known as the target of METTL14. METTL14 enhanced the m6A methylation of MIR155HG relying on the “reader” protein HuR-dependent pathway and then modulated PD-L1 expression, contributing to the immune escape of HCC cells (127). In intrahepatic cholangiocarcinoma (ICC), the loss of ALKBH5 enhanced m6A abundances of PD-L1 transcripts in the 3′-UTR region in a YTHDF2-dependent manner (132), downregulating its expression. A recent study demonstrated that PD-L1 expression is positively correlated with the expression of METTL3 and IGF2BP3 in breast cancer. Mechanistically, METTL3 upregulated PD-L1 expression by facilitating its mRNA stability in an m6A-IGF2BP3-dependent manner (123), which may guide new directions for breast cancer immunotherapy. Recently, PD-L1 expression has also been found to be regulated by some other m6A regulators in other types of cancer, such as mediated by METTL3 in OSCC (124), by FTO in colon cancer cells (131), and by YTHDF1 and YTHDF2 in NSCLC (136). Additionally, Liu found that the knockdown of IGF2BP1 downregulated PD-L1 expression and activated immune cell infiltration, thus leading to the inhibition of HCC progression (134).

#### m6A modification and other checkpoints

However apart from PD-1 and PD-L1, m6A regulates other checkpoints, such as CD80, ICOS, and VISTA. Consistent with PD-1 and PD-L1, METTL3 was found to promote the translational expression of CD80, which was identified as another novel checkpoint that promotes DCs activation and maturation in an m6A-dependent manner (125). Moreover, METTL3 has been reported to promote T follicular helper (TFH) cell differentiation through m6A modification. Meanwhile, the m6A level and expression of inducible co-stimulatory (ICOS) were decreased in METTL3-deficient TFH cells, indicating that m6A modification regulated ICOS expression (126). A similar study from Yangyang Zhu’s group reported that the decreased expression of ICOS, caused by glyceraldehyde 3-phosphate dehydrogenase protein weakened TFH cell initiation *via* METTL3/METTL14-mediated m6A modification (129). In CRC, YTHDF1 has been observed to enhance the protein levels of PD-L1 and VISTA (also known as PD-1 homolog (PD-1H), DD1α, Gi24), and the IgV domain of which shares a sequence homology with both CD28 and B7 families (140), in an m6A dependent manner (135). In addition to the aforementioned regulatory pathways validated experimentally, researchers have analyzed the relationship between m6A methylation modification and the expression of immunotherapeutic targets, such as PD-1, PD-L1, CTLA4, TIGIT, and LAG3 by bioinformatics, revealing the important roles of m6A modification patterns in the anti-tumor immunotherapy strategy for various cancers. For instance, the m6A score or m6A-related high/low-risk subgroup, based on the expression of m6A methylation-related genes, was characterized by differential expression of several immune checkpoints, including PD-1, PD-L1, and CTLA-4 in head and neck squamous cell carcinoma (141), bladder cancer (142–144), lung adenocarcinoma (145), clear cell renal cell carcinoma (146, 147), glioblastoma multiforme (148), HCC (149), gastric cancer (150), and small cell lung cancer (151). The aberrant expression of these immune checkpoints plays an important role in the efficacy of anti-PD-L1 immune checkpoint therapy, considered a predictor for the prognosis in patients treated with ICIs. However, these conclusions were assessed based on the dataset extracted from public databases and bioinformatics analysis, and clinical samples and cellular experiments are required to verify our findings.

#### m6A modification and other anti-tumor immunity regulating pathways

In addition to the effect on immune checkpoints, the response to anti-tumor immune therapy has been reported to be affected *via* reshaping the TME and regulating various signal pathways by m6A regulators. For example, depletion of Mettl3/14 strengthens the response to anti‐PD‐1 therapy by promoting interferon-gamma (IFN‐γ)‐Stat1‐Irf1 signaling in CRC and melanoma (152). Similarly, in melanoma, FTO knockdown increased tumor cell sensitivity to anti-PD-1 treatment by IFNγ in mice (153). Likewise, a recent study found that knockout of Alkbh5 inhibited tumor growth during anti–PD-1 treatment by regulating Mct4/Slc16a3 expression, lactate content, and suppressive immune cell accumulation in TME (37).

Furthermore, m6A modification was also reported to active and mature DC (125), promote more antigen presentation and CTL responses (154), increase infiltration of M1/M2-like TAMs and Treg cells (155), and suppress Tfh differentiation and maturation (126), all of which protect the homeostasis and tumor immunosurveillance function. For example, Yin’s group has reported that deletion of METTL3 in myeloid cells attenuated the effect of PD-1 blockade in B16 melanoma, and METTL3 KO mice showed increased infiltration of M1/M2-like TAMs and Treg cells in tumor sites compared to control mice (155). In a CRC-bearing mouse model, YTHDF1-deficient classical DCs presented a better therapeutic effect along with anti-PD-L1 treatment compared with that of the control mice by enhancing the ability of cross-presentation of tumor antigens of DCs and promoting the activation of T cells (154). Recently, Chen et al. demonstrated that METTL3 inhibited anti-tumor immunity by promoting the m6A-BHLHE41-CXCL1/CXCR2 axis to recruit immunosuppressive MDSCs suppressing CD8 + T cells, thereby facilitating CRC progression (156). Except for solid tumors, in AML, FTO inhibition has been reported to inhibit the expression of LILRB4, identified as an immune checkpoint gene that plays a vital role in the immunotherapy of AML, enabling AML cells to be more sensitive to T-cell toxicity and preventing immune escape, thus serving as a potential enhancer for immunotherapy of AML (157).

Aside from the remodeled TME described above, m6A modifcation mediated the anti-tumor immunity by affecting some tranditional pathways. Song et al. found that the deletion of METTL3 in tumor-infltrating NK cells is associated with malignant progression and shorter survival time in mouse models by downregulating SHP-2. Then, reduced SHP-2 made NK cells less responsive to IL-15, which is related to the inhibition of AKT-mTOR and MAPK-ERK signaling pathways (158). Recently, an analogous result has shown that METTL3-deficiency in macrophages destroyed the translation of SPRED2 mediated by YTHDF1, which promoted tumor growth and metastasis through the activation of ERK and NF-κB/STAT3 pathways (155).

Accumulating evidences have demonstrated that out-of-balance between host microbiota and immune system played a role in tumorigenesis and progression (159). For example, the composition of gut microbiome differed significantly between responders and nonresponders in melanoma patients receiving anti-PD1 therapy (46, 160). According to transcriptome map, the microbiome is a significant influence factor on host m6A modification (161). As a result, the link between gut microbes and the host m6A modification may open up new avenues for ICIs against cancer. This data identifies a correlation between m6A and synergistic effects on immunotherapy outcomes, making it a promising target for combination cancer immunotherapy.

### Prognostic values of m6A regulators for ICI treatment

Traditional therapies, radiotherapy, and/or chemotherapy have been the only treatment methods for locally advanced and metastatic cancers for decades, and their overall survival remains stagnant. Since the discovery of ICIs, major breakthroughs have been made in prolonging overall survival or progression-free survival in patients with advanced and metastatic cancer. Tumor immunotherapy was designed to suppress the binding of tumor PD-L1 to PD-1 on the T cell surface, then reactive the inactivated immune cells, and eventually realize the role of recognizing and eliminating tumor cells more effectively. Clinical data have shown that ICIs yield remarkable anti-tumor activity and produce durable, long-term responses with limited side effects, which rapidly turn into a promising cancer therapeutic approach in a subset of patients with various tumor types. There are different FDA-approved ICIs for various malignancies, including atezolizumab, durvalumab, nivolumab, pembrolizumab, avelumab, and ipilimumab. Targeting both co-stimulatory and coinhibitory T cell receptors, regarded as potential and clinical development of agents for immunotherapy, are also ongoing clinical trials, such as targeting LAG3, TIM3, TIGIT, and BTLA, as well as agonists of the co-stimulatory receptors GITR, OX40, 41BB, and ICOS (162).

Despite the significant clinical benefits, several limitations urgently need to be resolved, including low response, acquired resistance, and severe side effects (163). A significant difference in the level of expression of m6A regulators between responders and non-responders has been shown based on TISIDB, indicating its great potential for clinical application (**Table 4**). As can be seen from the form, differential expressions of FTO, YTHDF2, IGF2BP1, KIAA1429, HNRNPA2B1, ABCF1, FMR1, and IGF2BP3 were observed between the responders and non- responders for patients with urothelial cancer treated with atezolizumab. Similarly, a discrepancy in the expression level of IGF2BP1 was found in patients with MAPKi melanoma treated with pembrolizumab and nivolumab. In gliomas, the signature of nine m6A-related genes involved in immune response regulation has been identified by GO and KEGG analysis, despite the lack of clinical sample validation (164).These findings highlight the key role of m6A regulators in prediction of immunotherapy outcomes. Additionally, a major effort has been made to develop targeted drug-based m6A regulators for cancer immunotherapy to help develop personalized immune therapeutic strategies for non-responsive patients.

**Table 4:** The expression difference of m6A regulators in different cancer patients with ICI treatment.

m6A regulator	Cancer	Drug	Responder	Non-responder	P-value	PMID
FTO	Urothelial cancer	Atezolizumab(anti-PD-L1)	68	230	0.0191	29443960
YTHDF2	Urothelial cancer	Atezolizumab(anti-PD-L1)	68	230	0.026	29443960
IGF2BP1	Urothelial cancer	Atezolizumab (anti-PD-L1)	68	230	0.0295	29443960
IGF2BP1	Melanoma	Pembrolizumab Nivolumab (anti-PD-1)	6	5	0.0384	26997480
IGF2BP3	Urothelial cancer	Atezolizumab(anti-PD-L1)	68	230	0.0396	29443960
KIAA1429	Urothelial cancer	Atezolizumab(anti-PD-L1)	68	230	0.0373	29443960
HNRNPA2B1	Urothelial cancer	Atezolizumab(anti-PD-L1)	68	230	0.000267	29443960
ABCF1	Urothelial cancer	Atezolizumab(anti-PD-L1)	68	230	0.015	29443960
FMR1	Urothelial cancer	Atezolizumab(anti-PD-L1)	68	230	0.0224	29443960

### Targeting m6A regulators for cancer immunotherapy

Immunotherapy remains a promising treatment for cancer, but its low responsiveness remains. Based on the above studies, we have concluded that m6A modification has an important role in tumor immunotherapy. Developing inhibitors/agonists of m6A regulators is a promising therapeutic strategy for improving the immune response, and in combination with immunotherapy, may re-sensitize tumor cells to anti-cancer drugs (**Table 5**). Although inhibitors/agonists have not yet been widely used in clinical practice, they have shown promise in suppressing tumor growth in animal models of cancer.

**Table 5:** Effects of inhibitors of m6A regulators in combination with immunotherapy in various cancers.

Target	Name	Cancer	Mechanism
FTO	FB23-2	HCC	promote antigen presentation and TIDC maturation (165)
FTO	MA	Prostate cancer	promote CD8+ T proliferation and infiltration, and suppress the transcript of PD-L1 (166)
FTO	Dac51	Melanoma NCSLC	increase T cell infiltration (167)
ALKBH5	ALK-04	Melanoma	recruit immunosuppressive MDSCs and Tregs, and lactate accumulation (37)
METTL3	STM2457	CRC	increase activated T cells infiltration (156)

#### Targeting m6A erasers

Among the m6A regulators, FTO is the most promising target for the development of inhibitors, and to date, over 10 FTO inhibitors have been discovered, and their treatment efficiency has been verified in different models (168). Rhein, identified as the first FTO inhibitor, reversibly binds to the catalytic domain of FTO and inhibits the demethylation of m6A catalyzed by FTO with low cytotoxicity. Yan et al. demonstrated that in combination with Rhein and tyrosine kinase inhibitor (TKI), resistant cells were sensitive to TKIs and inhibited colony formation compared with that of the single-agent treatment in leukemia cells (169). Unfortunately, no clinical studies have reported the effect of the combination of rhein and immunotherapy on tumors.

Recent studies have shown that the combination strategy of antigen-capturing nanodrug and FTO inhibitor (FB23-2) showed tremendous potential to drive immune checkpoint blockade (ICB)-based immunotherapy, thereby assisting ICB in preventing distant tumor growth and metastasis after the primary tumor ablation therapy of HCC. FB23-2 was released intracellularly from the nanodrug and triggered a significant anti-tumor immune response by promoting antigen presentation and maturation of TIDC by increasing the m6A modification level (165). Meclofenamic acid, a highly selective FTO inhibitor, has been loaded into the γ-cyclodextrin cavity, which enhanced photothermal immunotherapy against PCa when combined with near-infrared radiation -II-mediated photothermal therapy based on gold nanorods. *In vivo*, the mice with the synergistic therapy showed greater tumor growth inhibition and significantly fewer pulmonary metastatic nodules than that of the control mice by promoting the proliferation and infiltration of CD8+ T cells in the TME and suppressing the transcript of PD-L1 (166).

Recently, a study reported that loss of FTO inhibited tumor growth and blocked FTO-mediated immune evasion by enhancing CD8+ T cell infiltration in tumors. Furthermore, a novel FTO inhibitor, Dac51, facilitated the effect of PD-L1 blockade therapy for better tumor control, showing slower growth and longer overall survival by increasing T cell infiltration, indicating a potent strategy to synergistically improve the immune response in melanoma and NSCLC (167). Consistently, Yang et al. reported that inhibiting FTO enabled the sensitivity of the response to anti-PD-1 therapy *in vivo* by mediating the regulation of PD-1, CXCR4, and SOX10 in melanoma (130), revealing a critical role of FTO inhibition in accelerating melanoma cell resistance to anti-PD-1 blockade (170).

Subsequently, Su et al. screened two highly effective and selective FTO inhibitors, CS1 and CS2, showing a better effect in suppressing leukemia cell activity than FB23-2 and MO-I-500 (two other previously reported FTO inhibitors). CS1/CS2 treatment restricted AML stem cell self-renewal and suspended immune escape by decreasing the expression of the immune checkpoint gene LILRB4 (157). And the combinatorial treatment of MA2, another inhibitor of FTO, plus the chemotherapy drug temozolomide(TMZ), exhibited a synergistic effect in suppressing glioma cells (171). Thus, developing m6A-based targeted drugs to improve cancer immunotherapy will occur in the near future.

In another case, ALK-04, a specific inhibitor of ALKBH5, functioned synchronously to accelerate anti-PD-1 treatment in melanoma, significantly decreasing tumor growth compared to control, by regulating the recruitment of immunosuppressive regulatory T cells and myeloid-derived suppressor cells, and lactate accumulation in the TME (37). It was also demonstrated that downregulation or mutation of ALKBH5 was correlated with a positive response to PD-1 therapy in patients with melanoma treated with pembrolizumab or nivolumab (37). In ICC, studies on specimens have revealed that patients with stronger nuclear enrichment of ALKBH5 are more sensitive to anti-PD1 immunotherapy (132). Therefore, ALKBH5 inhibitors/activators are potential therapeutic targets for improving the efficacy of immunotherapy.

#### Targeting m6A writers

METTL3/METTL14, two core catalytic subunits of MTC, have been reported to play a crucial role in tumorigenesis and the maintenance of CRC. A recent study identified that in combination with STM2457, a highly potent and selective catalytic inhibitor of METTL3, anti-PD1 treatment resulted in a remarkable anti-tumor effect compared with that of the individual treatments in CRC mouse models through the increased infiltration of activated T cells (156). As mentioned before, METTL3/14 knockout in CRC enhanced the response to anti–PD-1 therapy in constructed Patient-Derived Xenograft (PDX) mice by increasing the infiltration of cytotoxic CD8+ T cells into the tumor cell and a marked increase in the concentration of IFN-c, Cxcl9, and Cxcl10 in the tumor microenvironment (128). Similar to immunotherapies listed above, Li et al. showed that METTL3 was upregulated in glioblastoma patients with TMZ treatment, and silencing METTL3 inhibited the tumor growth in TMZ-resistant xenograft mouse in combination with TMZ (172).

Several different inhibitors/activators have also been reported to exhibit anti-tumor effects. For example, STM2457 targeted key stem cell populations of AML to reverse the malignant phenotype of AML, revealing significant anti-leukemic effects (173). A synergistic anti-cancer effect through the combination of STM2457 and immunotherapy is looking forward to going into clinical trials.

#### Targeting m6A readers

In line with the inhibitors of writer/eraser, a similar synergistic effect was observed when combined with anti-PD-L1 therapy. Han et. reported that when WT and Ythdf1 −/− tumor-bearing melanoma cells mice were treated with an anti-PD-L1 antibody, all Ythdf1−/− mice showed complete tumor suppression, whereas only 40% of the control group showed regression (154). Similar results were observed in CRC, mice-bearing YTHDF1-knockdown CT26 and MC38 tumor cells showed significantly reduced tumor growth rate and weight, and the overall survival of mice receiving the combination therapy was dramatically prolonged compared with those in either monotherapy group (135). MDNP/siYTH polyplexes, employed for the non-viral delivery of YTHDF1 siRNA into DCs, displayed the best tumor growth inhibition in the MDNP/siYTH polyplexes + aPD-L1 combinational treatment mice group (174). It uncovered YTHDF1 depletion that could potentiate the efficacy of anti-PD-L1 immunotherapy. Unfortunately, no corresponding inhibitor of YTHDF1 has been developed and applied to practice so far.

Accumulating evidence has demonstrated that the connection between the dysregulation of m6A regulators and the efficiency of ICIs treatment,which played an essential role in tumor progression. Thus, inhibitors/agonists of the m6A regulator protein can be applied as adjuvant therapies for immunotherapy to make ICI therapy safer and more effective. However, inhibitors/activators of m6A regulators have not yet been used for the treatment of cancer, and future clinical trials are required to prove the synergistic anti-cancer effect of m6A inhibitors combined with ICIs.

## Conclusions and prospects

Over the past few decades, ICI therapy has resulted in a leap forward in the treatment of various types of cancer and has achieved unprecedented improvement in overall and progression-free survival, especially in several advanced and metastatic cancers, along with many failing trials. However, due to intra-tumor and inter-tumor heterogeneity and the dynamic imbalance of immune biomarkers, many patients still fail to respond to immunotherapies or show resistance to ICIs treatment after the initial response. Therefore, there remains a need for an in-depth exploration of the mechanism of ICI resistance and the search for additional targets to make the best of the full benefits of immunotherapy.

In recent years, a large number of studies have shed light on the roles, underlying mechanisms, and possible therapeutic implications of m6 A-related regulators in a wide range of cancers. What is more, some m6A inhibitors have shown promising therapeutic efficacy with/without targeted therapy and chemotherapy drugs in the treatment of solid tumors and hematologic tumors. Unfortunately, no clinical trials on the effect of the combination of m6A inhibitors and immunotherapy on tumors have been published to date. We have summarized the recent advances in the design of combinatorial treatment with m6A inhibitors plus the ICIs, which demonstrated an improved immune response rate, reactivation of the response against anti-PD-1 blockade, and achieving a better tumor control. Additionally, we have reviewed the prognostic values of m6A-modification regulators for ICI therapy.

Herein, we generalized the mechanism of m6A modification and its regulators related to the progression and prognosis of various types of cancer by regulating key pathways. We also explored many abnormally expressed by m6A modulators that are deeply involved in the regulation of the expression of immune checkpoints, which influence the clinical responses to immunotherapy, and might be novel therapeutic targets in cancer treatment. Subsequently, several inhibitors targeted m6 A regulators have been discovered, which have wide implications in various cancers when combined with immunotherapy.

Many researchers have devoted themselves to uncovering the role of m6A regulators in critical pathological processes related to tumorigenesis and progress by regulating the epitranscriptome, opening a new window for cancer treatment. Wang et al. and Li et al. reported that m6A modification helped relieve resistance to anti-PD-1/PD-L1 therapy in patients with CCA. Depletion of Mettl3/14 and ALKBH5 in CRC cells enhanced the response to anti‐PD‐1 treatment in mice (37, 128). PCIF1, the writer of m6Am, has been shown to modulate anti-PD-1 therapy in CRC (35).Thus, the inhibitors/activators of m6A/m6Am regulators have shown potential clinical implications as adjuvant therapies to improve the response to anti-PD-1 therapy or reduce resistance.

Increasing investigations have elaborated that the “writers,” “erasers,” and “readers” displayed profound roles in the expression of immune checkpoints, such as PD-1, PD-L1, and CTLA4. Drugs targeting m6A regulators incorporated with anti-PD-1 treatment have shown remarkably enhanced effects in mouse models. Inhibition of “writers” (METTL3 or METTL14) and “erasers” (ALKBH5) enhanced the efficacy of anti-PD-1 blockade in CRC (37, 128). The possible reasons include the following (1): “writers” (METTL3 or METTL14) and “erasers” (ALKBH5) have the opposite trend of expression: one is upregulated, and the other is downregulated (2). Different targeted mRNA or ncRNAs: one is an oncogene, and the other is homeostasis (3); some unknown “writers” and “erasers.”

We have summarized the application of specific inhibitors of m6A regulators that inhibit cancer cell growth and accelerate apoptosis, and that, combined with R-2HG and other anti-cancer treatments, synergistically control leukemia progression in mice. Nevertheless, the potential side effects of m6A‐based inhibitors should not be ignored.

The dysregulation of m6A plays a key role in mediating the response to ICIs. Therefore, combining inhibitors of m6A regulators with immunotherapy seems to be an attractive and promising strategy to compensate for the limitations of immunotherapy. Although m6A inhibitor treatments for cancer are currently not used in clinical trials, it has pointed the right direction and paved the way for discovering an effective novel therapy for cancer treatment, which might transform the tumor into a chronic disease, permitting patients to survive and maintain their quality of life. However, m6A studies in various types of cancers are still incipient, and an in-depth understanding of the molecular mechanisms of m6A modification is warranted to fully elucidate the diverse aspects of m6A modification, which will provide vital information for individualized treatment. In the future, several new agents that may face great challenges provide more opportunities for patients with cancer.

## Author contributions

JP, ZD: Researched the topic, and wrote the manuscript. JP: Prepared the illustrations. TH and CZ edited the paper. All authors contributed to the article and approved the submitted version.
